# No changes in adolescent’s sedentary behaviour across Europe between 2002 and 2017

**DOI:** 10.1186/s12889-021-10860-3

**Published:** 2021-04-23

**Authors:** J. López-Fernández, A. López-Valenciano, X. Mayo, G. Liguori, M. A. Lamb, R. J. Copeland, A. Jiménez

**Affiliations:** 1 Centre for Exercise, Sport and Life Sciences (CSELS), Coventry University, Coventry, West Midlands UK; 2 GO fit LAB, Ingesport, Madrid, Spain; 3 Observatory of Healthy & Active Living of Spain Active Foundation, Centre for Sport Studies, King Juan Carlos University, Madrid, Spain; 4 The College of Health Sciences, University of Rhode Island, Kingston, RI USA; 5 Centre for Behavioural Science and Applied Psychology, Sheffield Hallam University, Sheffield, UK; 6 Advanced Well-Being Research Centre, Sheffield Hallam University, Sheffield, UK; 7 The National Centre for Sport and Exercise Medicine, Sheffield, UK

**Keywords:** Sedentarism, Sitting, Youth, National policies

## Abstract

**Background:**

Public health organizations have been alerted to the high levels of sedentary behaviour (SB) among adolescents as well as to the health and social consequences of excess sedentary time. However, SB changes of the European Union (EU) adolescents over time have not been reported yet. This study aimed to identify SB of the EU adolescents (15–17 years) in four-time points (2002, 2005, 2013 and 2017) and to analyse the prevalence of SB according to the sex.

**Methods:**

SB of 2542 adolescents (1335 boys and 1207 girls) as a whole sample and country-by-country was analysed in 2002, 2005, 2013, and 2017 using the Sport and Physical Activity EU Special Eurobarometers’ data. SB was measured using the sitting time question from the short version of the International Physical Activity Questionnaire (IPAQ), such that 4h30min of daily sitting time was the delineating point to determine excess SB behaviour (≥4h30min of sitting time) or not (≤4h30min of sitting time). A χ2 test was used to compare the prevalence of SB between survey years. Furthermore, SB prevalence between sexes was analysed using a Z-Score test for two population proportions.

**Results:**

The prevalence of SB among EU adolescents across each of the four survey years ranged from 74.2 and 76.8%, rates that are considered high. High levels of SB were also displayed by both sexes (girls: 76.8 to 81.2%; boys: 71.7 to 76.7%). No significant differences in the prevalence of SB among years (*p* > 0.05) were found for the whole sample, and for either girls or boys. Also, no significant differences in the prevalence of SB between girls and boys were found.

**Conclusion:**

The SB prevalence in European adolescents is extremely high (76.8% in 2017) with no differences between girls and boys. No significant improvements have been seen between 2002 and 2017. Eurobarometer should increase the adolescents’ sample to make possible benchmarking comparisons among the EU countries and extend the survey to the younger children population.

## Background

Sedentary behaviour (SB) represents those behaviours performed in sitting or lying position with a low level of energy expenditure (≤1.5 metabolic equivalent of tasks [METs]) [1]. In adolescents, these behaviours represent between 60 and 70% of daily time spent awake [2–4]. SB has become one of the main risk factors for weight and adiposity weight [5–7], psychological health problems (e.g., anxiety, depression, aggression, attention problems) [8, 9], and is also seen as increasing the vulnerability to suicide in adolescents [10]. Furthermore, evidence concludes that SB adopted during adolescence will be maintained into adulthood [11] and it is therefore a strong predictor of cardiovascular diseases later in life [12, 13].

Although there is no sufficient evidence for SB’s determinants in young people [14], adolescents spend prolonged periods of their awake time sitting in controlled, required, environments such as schools [15, 16], which thereby hinders the possibility of limiting SB time. Alongside this, the electronic revolution has transformed people’s movement patterns, significantly increasing the amount of daily time in front of the screen (e.g., televisions, computers, smartphones, etc.) [17, 18], and by sex, girls usually report a slightly higher prevalence of SB than boys [3]. Furthermore, there seem to be sex differences in how SB is accumulated, with boys reporting more screen time (televisions or computer games) and girls spending more time in communication-based SB (surfing the internet, texting, talking on the phone) [17, 18]. For those reasons, concerns among parents, health care professionals, governments, educators, and researchers about SB’s effects on young people’s health have increased.

Despite this, to our knowledge, by the time this paper was written, there was not a European guideline or policy about SB in adolescents. It was not until 2020 when the World Health Organization (WHO) included the first SB recommendations within their guidelines for adolescents (“limit the amount of time spent being sedentary, particularly the amount of recreational screen time” [19]). There was a previous SB guideline published by the WHO but, for children under 5 years old [20]. It was not until 2011 when the first national SB guideline for children and adolescents was published, in Canada [21], providing an important and timely recommendation for advancing of SB public health agenda. Regarding the EU, despite the recommendations to reduce SB in school-age children from the EU Physical Activity Guidelines in 2008 [22], only a few countries (e.g., Germany, France, Spain, or United Kingdom) have included some actions since then to reduce SB in their national guidelines [23–25]. However, none of these guidelines have included sex-related recommendations [26]. Furthermore, most of the reports about PA from the EU countries, which include an evaluation of the SB indicators about the compliance of the no more than 2-h screen time recommendations, show poor compliance with the existing guidelines [27]. Therefore, organizations and governments should place a greater emphasis on reducing SB during adolescence [28] through the establishment of guidelines and policies with specific goals and key performance indicators, and this should be done with consideration to sex-based differences in SB [11, 29].

Based on this, it is relevant to monitor the SB of European adolescents across different time-points. This is especially important since the WHO’s Global Action Plans emphasises the need to implement effective and coordinated actions aiming to reduce SB for both adults and children [30, 31]. However, the lack of studies monitoring the prevalence of SB prevents the establishment of a baseline, therefore determining long-term objectives and success [30, 31]. The Special Eurobarometer, in which the International Questionnaire of Physical Activity (IPAQ) is administered, might be a good opportunity to identify this baseline point in the EU and for analysing the effect of future policy development on SB in the mid and long term. In fact, the IPAQ questionnaire asks about daily sitting time, which has proved to be useful for analysing the prevalence of SB in European adults and for evaluating over different time periods [32, 33].

This study aimed to analyse the SB prevalence in EU adolescents (15–17 years) between 2002 and 2017, considering data from the four separate *Sport and Physical Activity* Eurobarometer’s data. A secondary objective of this research was to compare the prevalence of SB according to the sex.

## Methods

### Data source

All methods were carried out in accordance with relevant guidelines and regulations. The European Commission conducts public opinion surveys simultaneously on all EU state members to identify the levels of PA, sports participation, and SB among its citizens through the *Sport and Physical Activity and Health and Food Special Eurobarometers*. These Eurobarometer surveys were conducted using a multi-stage sampling, random design. In order to cover the whole territory of the country, the number of sampling points was drawn with probability proportional to both population size and population density.

For the purposes of this study, data from adolescents (15–17 years old) were obtained from four successive Eurobarometer surveys, December 2002 (Special Eurobarometer 183.6; *n* = 543), December 2005 (Special Eurobarometer 246; *n* = 929), December 2013 (Special Eurobarometer 412; *n* = 592), and December 2017 (Special Eurobarometer 472; *n* = 478), with a final sample of 2542 adolescents (1207 girls and 1335 boys) from the 28 European Union member countries (Austria, Belgium, Bulgaria, Czech Republic, Croatia, Cyprus Republic, Denmark, Estonia, Finland, France, Germany [combined West and East Deutschland], Great Britain, Greece, Hungary, Ireland, Italy, Latvia, Lithuania, Luxembourg, Malta, Netherlands, Poland, Portugal, Romania, Slovakia, Slovenia, Spain, and Sweden). Data from Northern Cyprus and Turkey were not analysed because they do not belong to the EU member countries. Following the methodology used in previous studies using Eurobarometer data, Northern Ireland was not considered because the sample size from this region was too high compared to the sample from the UK [32].

### Measures

The IPAQ is a valid and reliable questionnaire to obtain data on SB [34]. The IPAQ short form records PA at three intensity levels along with the total sitting time on an average day (i.e., *How much time do you spend sitting on a usual day? This may include time spent at a desk, visiting friends, studying, or watching television?*). In the 2002 and 2005 surveys, participants were asked to estimate their usual weekday sitting time using an open-ended response scale. On the contrary, for the 2013 and 2017 surveys, participants were given a choice of 11 categorical response options ranging from ‘≤ 60 mins’ to ‘>8h30mins’.

For this study, surpassing the cut-off point of 4 h and 30 min of sitting time was considered as SB. This value was based on the cut-off point for increased risk of cardiovascular diseases [35, 36]. Individuals answering “don’t know” on the sitting question were removed from the analysis.

### Statistical analysis

Descriptive statistics presented as a proportion (%) with the 95% confidence interval (95% CI) were calculated for the SB dichotomic variable. The χ2 test was implemented for studying the association between sedentary lifestyle (SB and non-SB) with the studied years (2002, 2005, 2013, and 2017). Due to the number of EU countries increasing from 15 to 28 in 2004, two analysis were performed. The first analysis compared outcomes from 2002 to 2017 considering data from all countries participating in each Special Eurobarometer. The second analysis also compared the outcomes from 2002 to 2017 but only considering the data from the first 15 countries [32]. The differences by sex in SB for each studied year were analysed using a Z-Score for two population proportions. A priori alpha level was set at 0.05. Z-score analyses were performed with Microsoft Excel version 1709 (Microsoft Corporation; Redmond, Washington, United States of America). The remaining analyses were performed using the Statistical Package for Social Sciences (version 22.0, SPSS Inc., Chicago, IL, USA).

## Results

Table 1 displays the descriptive outcomes for non-SB and SB among the studied years for each of the analysed countries. The rates of SB across the four survey years were high as they ranged from 74.2 and 76.8%. High levels of SB across these four years were also displayed by both sexes (girls: 76.8 to 81.2%; boys: 71.7 to 76.7%).

**Table 1:** Prevalence (%) of sedentary behaviour (SB) in adolescents (15–17 years old) in the European Union (EU) countries between 2002 and 2017

	2002			2005			2013			2017			2002–2017	
	Sample	SB (***%)***	95% CI	Sample	SB (***%)***	95% CI	Sample	SB (***%)***	95% CI	Sample	SB (***%)***	95% CI	χ2	***p***-value
EU total	*543*	74.2	70.2–77.7	*929*	78.8	76.2–81.4	*592*	78.0	74.5–81.4	*478*	76.8	73.0–80.7	4375	0.224
EU boys	*276*	71.7	66.3–77.5	*490*	76.7	72.7–80.2	*310*	75.2	70.3–79.7	*259*	75.7	70.3–80.7	2415	0.491
EU girls	*267*	76.8	71.9–82.0	*439*	71.1	77.2–84.7	*282*	81.2	76.2–85.8	*219*	78.1	72.6–83.1	2671	0.445
*Countries*														
Austria	42	66.7	52.4–81.0	21	66.7	47.6–85.7	13	76.9	53.8–100	9	88.9	66.7–100	2170	0.538
Belgium	40	85.0	72.5–95.0	36	88.9	77.8–97.2	30	76.7	60.0–90.0	16	68.8	43.8–87.5	3877	0.275
Bulgaria				44	84.1	72.7–93.2	19	68.4	47.4–89.5	20	80.0	60.0–95.0	2004	0.367
Croatia				31	71.0	54.8–87.1	10	80.0	50.0–100	13	92.3	76.9–100	2448	0.294
Cyprus Republic				28	89.3	78.6–100	16	81.3	62.5–100	15	60.0	33.3–86.7	5205	0.074
Czech Republic				32	87.5	75.0–96.9	20	85.0	65.0–100	12	83.3	58.3–100	0,147	0.929
Denmark	11	81.8	54.5–100	17	100	100–100	18	88.9	72.2–100	15	93..3	80.0–100	3225	0.358
Estonia				44	95.5	88.6–100	19	84.2	63.2–100	19	63.2	42.1–84.2	11,106	0.004
Finland	46	84.8	73.9–95.7	34	85.3	73.5–97.0	16	87.5	68.8–100	16	75.0	50.2–93.8	1169	0.760
France	25	64.0	44.0–88.0	26	88.5	73.1–100	20	100	100–100	24	70.8	54.2–87.5	11,424	0.010
Germany	46	76.1	63.0–87.0	43	88.4	76.8–97.7	34	76.5	61.8–91.2	15	86.7	66.7–100	3023	0.388
Great Britain	26	57.7	38.5–76.9	24	58.3	41.7–75.0	27	55.6	37.0–74.1	18	72.2	50.0–88.9	1428	0.699
Greece	34	76.5	61.8–91.2	21	90.5	76.2–100	32	68.8	53.1–83.1	31	77.4	61.3–90.3	3403	0.334
Hungary				25	84.0	68.0–96.0	15	93.3	80.0–100	5	60.0	20.0–100	3180	0.204
Italy	32	90.6	81.3–100	30	66.7	46.7–83.3	10	60.0	30.0–90.0	3	33.3	27.2–100	9170	0.027
Ireland	57	73.7	61.4–84.2	29	75.9	58.6–89.7	28	67.9	50.0–85.7	19	78.9	63.2–94.7	0,833	0.842
Latvia				37	76.1	65.7–86.6	32	81.3	68.8–93.8	29	72.4	55.2–86.2	0,677	0.713
Lithuania				48	72.9	60.4–85.4	43	81.4	69.8–93.0	15	80.0	60.0–100	1001	0.606
Luxembourg	21	95.2	85.7–100	29	93.1	82.8–100	20	100	100–100	19	89.5	73.7–100	2172	0.537
Malta				15	86.7	66.7–100	4	100	100–100	8	100	100–100	1728	0.421
Poland				50	84.0	72.1–94.0	13	69.2	38.5–92.3	15	80.0	60.0–100	1456	0.483
Portugal	53	43.4	30.2–56.6	32	40.6	25.0–59.4	16	50.0	25.0–75.0	20	45.0	25.0–69.9	0,396	0.941
Romania				40	42.5	27.5–57.5	23	47.8	30.4–69.6	31	71.0	54.8–87.1	6037	0.049
Slovakia				15	66.7	46.7–86.7	20	95.0	85.0–100	3	100	100–100	5786	0.055
Slovenia				45	88.9	80.0–97.8	17	76.5	52.9–94.1	26	84.6	69.2–96.2	1522	0.467
Spain	59	72.9	61.0–84.7	35	65.7	51.4–80.0	23	56.5	39.1–73.9	22	59.1	36.5–77.3	6640	0.451
Sweden	12	91.7	75.0–100	32	90.6	78.1–100	19	94.7	84.2–100	2	100	100–100	0,459	0.928
The Netherlands	39	84.6	71.9–94.9	36	80.6	66.7–91.7	35	97.1	91.4–100	38	94.7	86.8–100	7138	0.068

*CI* Confidence intervals

**Fig. 1:**
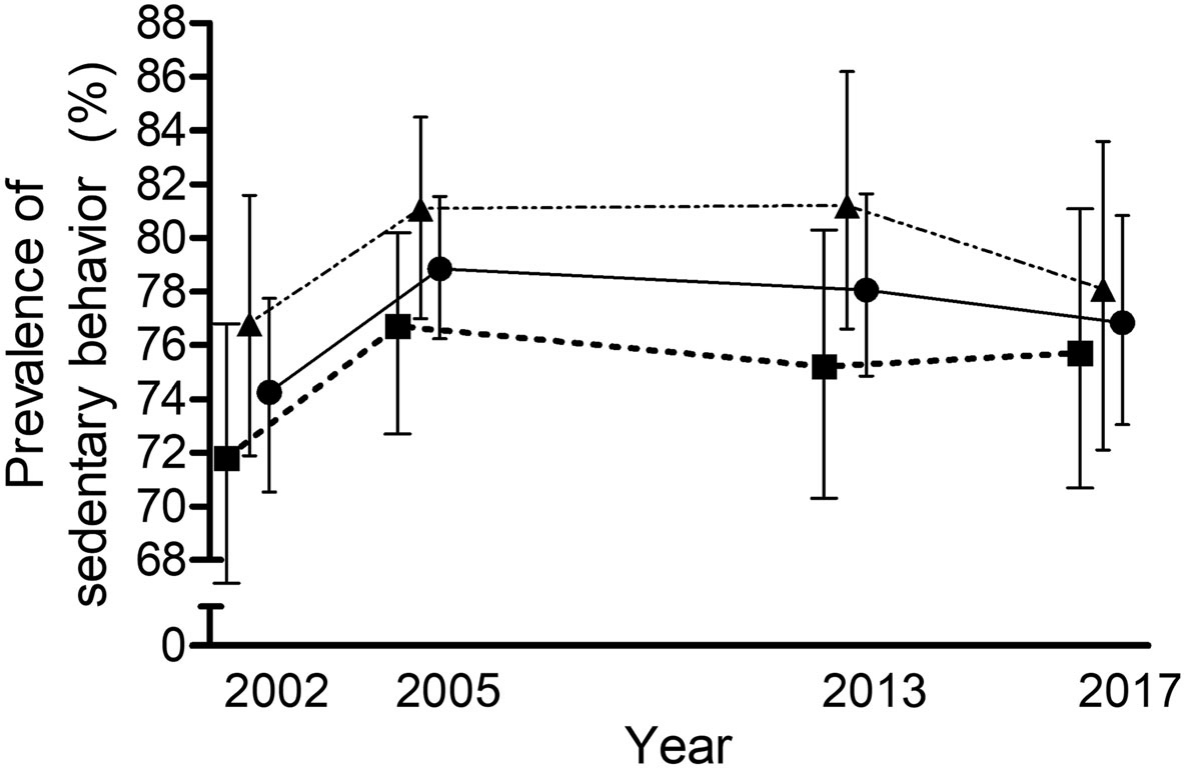
Prevalence of sedentary behaviour among European Union adolescents for the four studied time-points Prevalence (%) of sedentary behaviour (>4h30min/day) among European Union adolescents (in circles, the whole sample; in triangles, the girls’ sample; and in squares, the boys’ sample) for four different time-points (2002, 2005, 2013, and 2017). Data are means ± CI

No significant differences among years were found in the prevalence of SB for the whole sample (*n* = 2542; χ2 = 4375; DF = 3; *p* = 0.224), for girls (*n* = 1207; χ2 = 2671; DF = 3; *p* = 0.445) or for boys (*n* = 1335; χ2 = 2415; DF = 3; *p* = 0.491) (Fig. 1). The outcomes considering the first 15 EU countries did not reveal differences throughout the studied time points for either the whole sample [*n* = 1596; χ2 = 2665; DF = 3; *p* = 0.446], for girls [*n* = 762; χ2 = 2553; DF = 3; *p* = 0.466], or for boys [*n* = 834; χ2 = 1280; DF = 3; *p* = 0.734]). No differences in the prevalence of SB between girls and boys were found in the studied time point (2002 [Z-Score = 1.33; *p* = 0.18]; 2005 [Z-Score = 1.64; *p* = 0.10]; 2013 [Z-Score = 1.76; *p* = 0.08]; 2017 [Z-Score = 0.62; *p* = 0.53].

## Discussion

This study examined the data from the existing Eurobarometer reports in order to analyse how the SB prevalence in European adolescents has changed over 15 years (2002–2017) and if differences between girls and boys existed. The main findings were that (a) although EU adolescents showed high levels of SB, the prevalence of SB between 2005 and 2017 remains similar (74.2 to 76.8%; *p* > 0.05) with no significant differences over time for girls or boys; (b) girls and boys show similar prevalence of SB in all studied years.

Previous research has assessed the prevalence of adult SB across European populations based on 2002, 2005, 2013 and 2017 Eurobarometer data [32, 33, 37] but, to the best of our knowledge, this is the first study focused on adolescents. The Global Matrix project can be used to identify the percentage of European adolescents exceeding the 2-h of recreational screen time per day [27]. However, the total daily SB performed by European adolescents was still missing, and this research provides an initial approach to mend this gap. A high proportion of European adolescents, 76.8% in 2017, reported sitting times in excess of 4h30min, which is the threshold for SB. These rates of SB are higher than what has been reported for adults from the Eurobarometer data sets [32]. Unlike adults, however, this study did not reveal significant differences by sex in the prevalence of SB [32].

Although secondary-school is compulsory until the age of 16, most adolescents still attend secondary-school centres or other educational centres until the age of 18, so their time there may account for the high percentage of SB in adolescents. Thus, although some exceptions might exist (physical education classes, laboratory work, fieldwork, some technology or art classes, etc.), adolescents at school tend to accumulate more than 5 h of SB just during a typical school day [38]. This may also at least partly explain the lack of differences across the years, the lack of difference between sexes, and the higher prevalence of SB of adolescents compared to adults. After-school activities may also play a significant role in the total sitting time accrued, as adolescents spend an average of 59% of their after-school time in sedentary activities (from 27.7 to 88.9%) and screen-related activities usually represent the main sedentary activity [39]). Thus, to develop and analyse the impact of future policies and interventions addressing SB in adolescents, a distinction between sedentary behaviour at school and in free time should also be made. Furthermore, they type of sedentary activity should be also considered (e.g., screen-related activity; educationally related; socially related activity; etc.). These two considerations cannot be made through the IPAQ questionnaire, so a different instrument might be needed.

Regarding sex differences, most existing studies with adolescents suggest that girls accrue higher average sitting time than boys [38, 40–42]. The findings from our study are not in line with this, as no sex differences were seen in any of the study years. The lack of differences may be due to SB being self-reported, as opposed to more objective data such as from an accelerometer [33, 37]. Another possible explanation of the lack of sex differences might be the low sample size in the Special Eurobarometers as previous studies reported that girls and boys engage differently in sedentary activities, with boys reporting more TV or computer games, and girls reporting more time in communication or social media activities [17, 18]. Thus, further studies are needed to verify or reject the findings reported in our study.

To the best of the authors’ knowledge, only seven of the 28 EU countries (Austria, Belgium, Finland, France, Germany, Spain and The UK) included some kind of reference for sedentariness for children and youth in their national guidelines before publication of the latest Eurobarometer report (2017) [23–25, 43–47]. This is in contrast to the EU Working Group “Sport and Health” recommendations in 2008 to reduce SB in school-age children [22]. Moreover, the existing reports about PA from the EU countries show a high percentage of adolescents exceeding 2-h of screen-based entertainment per day [27], but do not monitor the daily sitting time of this same population. Since 2017, more European countries have developed or updated national guidelines related to SB in adolescents (Greece, Dutch, Latvia or the UK [48–51]). Nonetheless, other than recommendations like that exist in France (“*children between 6-17 years old should not accumulate sitting bouts for > 2-h long*”) [44], most other existing guidelines only mention SB under a qualitative perspective [43, 45–49], with quantitative recommendations mainly focused on screen-related activities.

### Limitation and strengths

This study has some limitations to be acknowledged: (a) Less than 600 adolescents were reported in three of the four Eurobarometer reports, so findings should be analysed carefully; in this regard, the sample size from each country is that small (varies between 13 to 58) that they should not be used to set the prevalence of SB for a given EU country in a particular year. Thus, benchmarking comparisons among countries is not possible either [32, 33], while no data were available for adolescents under 15 years; (b) SB was measured by a single self-reported question that is included within the IPAQ questionnaire, which is likely to underestimate the sitting time of adolescents [52]. However, as suggested with older adults, the use of the IPAQ Short form in this study should be valid as we compare groups within and between years instead of on individual basis [53]; (c) it is important to note that the sitting question of the IPAQ short version from 2002 to 2005 was an open solution of the total sitting time in a weekday, whilst, from the 2013 onwards the possible answers were closed to several categorical response options [54]. Finally, the existing reports do not distinguish between SB pattern or where they occur (at the educational centre or out the educational centre). Thus, future Eurobarometer surveys might consider making an extra effort to 1) get enough representation to allow both benchmark comparisons among European countries and strengthen the comparison analysis between girls and boys; 2) target other children population (i.e. pubertal, prepuberal or young children); 3) monitor the engagement on the most common sedentary activities for each under 18 years old group and be able to collect SB patterns; 4) monitor the sitting behaviour either at or out the educative centre.

Despite these limitations, it is important to consider that it is the first work that assess the prevalence of SB in European Union adolescents among four different time-points and provides an initial approach to the studied research question. It is expected that this initial approach provides a significant insight for European researchers, guideline developers, and policy makers in developing new strategies to address SB among European adolescents. Finally, this work has identified some limitations in Eurobaromenter reports that might be relevant to be addressed in future reports (e.g., low sample size or only adolescents are being monitored).

## Conclusions

European adolescents show worrying levels of SB regardless of their sex and no improvements have been achieved between 2002 to 2017. Likewise, girls and boys reported similar values of SB. European policy should develop guidelines to reduce this prevalence and set a common SB reduction target. Finally, European commission should increase the adolescents’ sample in the Eurobarometer reports to make possible benchmarking comparisons among the EU countries and extend the survey to younger children population.

## Data Availability

The raw data is owned by the European Commission and available online (Special Eurobarometer 183–6, December 2002: https://dbk.gesis.org/dbksearch/sdesc2.asp?no=3886&search=58.2&search2=&field=all&field2=all&DB=e&tab=0&notabs=&nf=1&af=&ll=10. Special Eurobarometer 246, December 2005: https://dbk.gesis.org/dbksearch/sdesc2.asp?no=4415&search=64.3&search2=&field=all&field2=&DB=e&tab=0&notabs=&nf=1&af=&ll=10. Special Eurobarometer 412, March 2014: https://dbk.gesis.org/dbksearch/sdesc2.asp?no=5877&search=Physical%20fitness%20and%20exercise&search2=&field=all&field2=&DB=e&tab=0&notabs=&nf=1&af=&ll=10. Special Eurobarometer 472, March 2018: https://dbk.gesis.org/dbksearch/sdesc2.asp?no=6939&search=Physical%20fitness%20and%20exercise&search2=&field=all&field2=&DB=e&tab=0&notabs=&nf=1&af=&ll=10).

## Abbreviations

CI: Confidence interval
EU: European Union
IPAQ: International Physical Activity Questionnaire
METs: Metabolic equivalent of tasks
SB: Sedentary behaviour
UK: United Kingdom
WHO: World Health Organization
